# Dental Prosthesis

**Published:** 1882-12

**Authors:** C. M. Wright


### THE DENTAL REGISTER.

VOL. XXXVI.] DECEMBER, 1882. [No. 12.

### COMMUNICATIONS.

### Dental Prosthesis.

BY C. M. WRIGHT, D.D.S.

[Read before the Mad River Dental Society.]

GENTLEMEN:—I have been requested to offer on this occasion, a few thoughts on the subject of dental prosthesis; although it has been called "thread-bare" for many years. Perhaps, for this very reason, we should be willing to give it more attention than it has received for some time—to cast away the rags and "thread-bare" covering, and rehabilitate it in proper garments. The occasion, too, of the resurrection of the "Mad River Valley Dental Society" may be a most appropriate one for this casting off of the old, and putting on of the new and better ideas, about this part of our Art. There is a Science of Medicine and there is an Art; and it is possible for a scholar to be deeply interested and highly cultivated in the Grand Science without pretending to be even a tyro in the Art. He may have spent years in his laboratory in the investigation of the supposed origin of disease, without having the slightest skill in detecting or distinguishing the simplest malady at the bedside. He may, in short, be a deep student of the science, without being an expert in the art. The science and the art are to this extent divided. A physician might, in his leisure hours, acquire an intimate knowledge of the Science of Dentistry; he might become expert in making preparations of dental tissues for histological studies, for the investigation of the theories in regard to dental caries; he might become well versed in a knowledge of the general pathological condition of the teeth, and yet, if called to the dentist's operating chair, could not diagnosticate an incipient alveolar abscess, nor an inflamed pulp, nor a pulp chamber filled with gas; not to mention the excavating of a carious cavity, and replacing with any dentists' materials. He does not possess the ART OF DENTISTRY.

The science of dentistry is a part of the science of medicine. I wish to say this distinctly and dogmatically. It is a fact not affected by the disputes of doctors or dentists. The Science of Dentistry is a branch of the Science of Medicine in its broadest signification. It springs from the same root; is nourished from the same fluid that circulates in the parent trunk, and all the other branches. I refer only to the SCIENCE, so called, and not at all to the ART. Here we must make a distinction. The Art of Medicine and the Art of Dentistry have but very little in common, as generally practiced in this country. And the art itself is so distinct from the science in dentistry and medicine, that men do become skillful and successful doctors and dentists with but a limited capital of general science—but, in its stead, cultivated perceptions and trained fingers. The young graduate of several native and foreign schools and universities may really possess very much more knowledge of what is called the Science of Medicine than the old practitioner, and yet, you and I would prefer to trust the man of ART, rather than the man of SCIENCE. The old practitioner, if possessed of natural talent (and this, however important, is like beauty—possessed only by the few, tho' desired by the many), has his senses cultivated to the point of intuition. He sees at a glance —he arrives at a correct conclusion instantaneously—by an unknown and unregarded mental process, that only experienced observation, practiced judgment, cultivation and habit can give. The *Art* is the important part. The Science has no other object, finally, than the practice of the art. In a country where no disease existed, the Science of Medicine would have no higher signification than the science of billiards. It would only be a pastime if men pursued it. It is because men are liable to disease, and demand the art of medicine, the art of dentistry, that the wisest men, for thousands of years, have given their lives to the study of the Science of Health and Disease. The curing of consumption is the ultimate object of Koch, of Berlin, in his curious studies and experiments with the germs of tubercle—even if he himself never expects to see a patient. His pupils, and all the world of doctors, must modify their *treatment* when his experiments are established. His Science will be felt in their Art. And so it is in Dentistry. Scientific investigation will modify our art. Science and art should walk hand in hand. Science advising—but art doing. Thus far, I am sure you will all agree with me; but can you go with me when I say that we have paid too much attention, lately, to Science—that we have held on to Science and neglected Art—that, in our admiration for Science—in our desire to be considered as members of a Scientific Profession—in our hope for a recognition by the Medical Profession, we have foolishly neglected our ART. We have let go the meat that we held in our mouths, to catch at the meat that we saw reflected in the water; and we have been in danger of losing the reality for the picture. The many articles on Dental Education that swamp our floating literature, show the tendency of the times. It is, I think, a sound proposition, and one that can, without much ingenuity, be perfectly defended, that "Knowledge that cannot be applied, is worthless, or no knowledge." If Knowledge is Power, it must be applied to exhibit itself. We have fallen down and worshipped knowledge, but have not had very clear ideas about what knowledge is, and have not cared to apply it. On the contrary, for many years it has been rather a term of reproach, very much employed by some of our best men, toward men who applied knowledge simply. For twenty years, I, myself, have heard on all sides, among my brethren, such condemnations:—"Dr. A——— is a good fellow, yes; but the fact is, he is only a mechanic." "Dr. B——— is a good gold filler, and that is all." Now, when I hear such things from prominent members of the Dental Profession,—men whose judgments I regard—whose opinions I respect,—what must I think? The inference is plain—that for a dentist to be a mechanic, is not the proper thing—the mechanic lowers the status of his profession. These are American views. I might say modern American views, for the fathers were not so foolish. When a scientist like OWENS, and before the congregated dentists of the world, can honestly compliment the dental profession—as Dr. Taft, and I heard him, in London, at the World's Medical Congress—on the excellence of the mechanical skill that had furnished him with a denture that gave him as little discomfort as the natural organs, and performed their fnnctions to all intents and purposes as well, we need not blush at possessing mechanical skill. When we can save *easily* and *comfortably* a natural denture by our handiwork, by our mechanical skill in cutting out caries and substituting gold or tin, we are applying the science to the ART of Dentistry. The ART, from the diagnosis to the last polish of a filling, or the extracting and inserting of a plate, is all mechanical. The ART OF DENTISTRY is what dentists propose to practice. The art of dentistry is a mechanical art, and can only be well practiced by a mechanic. Skilled fingers are a *sine qua non* in successful dental practice. We are mechanics. Our profession is a mechanical profession. Dental therapeusis is mechanical contrivance. And yet, brethren, American dentistry, with all the boasted excellence—with all the self-laudation—with all the assurances that she has heaped upon herself—to use a slang expression—with all her *cheek*—has, in the department of Dental Prosthesis, sadly degenerated. We are a profession of half-trained mechanics. We are bunglers. Not one in twenty practitioners (not students or graduates), but practitioners, are skillful mechanics, either in their laboratories or in their operating rooms. The nineteen have never trained their fingers—they have never given their days and nights to practicing to acquire *skill*, expertness, neatness, handiness. They bungle, from the taking of the impression to the finishing of a rubber plate. Now, if you will not accept this statement, which I firmly believe, and base my belief on my observation, and reasoning from the teachings of prominent men in regard to this branch. I say, if you will not believe this, perhaps, you must believe that as far as "art and beauty" are concerned, as far as the selection and arrangement of teeth, and regard for plastic anatomy are concerned, in the Prosthetic branch, we have, as a nation, about as little cultivation as you would expect to find in a Sioux Indian—or Buffalo Bill. In every street car—in every church—in every assembly—in every place—in short, wherever you go in these dear United States, where our so-called civilization exists, you find mouths disfigured by dentists. You do not notice it so much, because, like the Indian who gets used to seeing war-paint, you rather admire it, but cultivated foreigners, other and civilized strangers, and Americans who have lived in England, France, Switzerland or Germany, do notice it, from the time they land in New York till--well, till they, too, get used to it--that the disfigurement of the mouth and face by American dentists is a terrible national calamity. We disfigure with gold to a sickening extent. We disfigure with porcelain to a criminal extent. We get other vandals in the profession to disfigure us. I have talked to dentists, within a week, whose teeth, upper and lower, are so bespattered with gold that I could not keep my eyes off of them, and although I have been intent on the sight, to the exclusion of any *expression* of the mind that might have played about their lips, yet, when a laugh has suddenly displayed the whole effect of the bespangled teeth, I have shuddered at the ghastly spectacle. In the American Dental Association that met last August, the display of china, or bits of white marble, arranged regularly under the lips of several elderly and prominent members, was only another proof to me that we are but little better than barbarians, as far as cultivation of taste goes. I am not saying all this simply to make a sensation, or to keep up my reputation of writing in a pyrotechnic style. I am in the most selemn earnest possible. I feel it in my soul--this disgrace--this glaring fault of a profession that I love--of a class of men that form my only friends, on this and the other side of the Atlantic. Dear brethren; you have known me from boyhood, and you know that I would not bring such a charge against the profession unless I believed it from the very depths of my heart. Is there no remedy? No hope for the future? I think there is. And as I have made the accusation, I would beg leave to suggest a remedy.

*First*—Let us get a little card printed, or neatly written, and place it on our operating tables, so that every time we pick up an instrument it shall saucily stare us in the face—the printing or the writing should say: "The highest Art of the Dentist is to conceal his Art."

*Second*—Let us sit down and think of how much there is to be learned in the department of dental Prosthesis; and remember how many students start out in practice after only two or three years study; and let us adopt the English way of taking apprentices to our trade. How you shudder at the idea; but, remember, I shudder at your horrible disfigurement of human faces by your unskillful work. Yes, take apprentices; and let them begin early, and work at the trade of dentistry.

*Third*—Do not use teeth in sets from the combination or any other factories. The dental dealers clothe the American's mouths with their wares. Pick up odd teeth, cut them down, grind them, break them, repolish, or, reglaze in your furnaces. Keep your apprentices busy. Try to give each tooth an expression. If you can do this after you have practiced the mechanical art in your work-shop, you will begin to be respected as artists. You will begin to gain reputation. You will begin to receive a $100 from the rich when now you grudgingly receive $10. You will add lustre to the name of American dentistry. It has a little lustre, now, but it is rapidly getting dull and rusty; and will soon be cast aside entirely, among the nations.

